# Reply to Nwosu, C.; Ward, K.D. Comment on “Alharbi et al. Adoption of Health Mobile Apps during the COVID-19 Lockdown: A Health Belief Model Approach. *Int. J. Environ. Res. Public Health* 2022, *19*, 4179”

**DOI:** 10.3390/ijerph192416915

**Published:** 2022-12-16

**Authors:** Nouf Sahal Alharbi, Amany Shlyan AlGhanmi, Mochammad Fahlevi

**Affiliations:** 1 Department of Health Administration, College of Business Administration, King Saud University, Riyadh 11451, Saudi Arabia; 2 Department of Health Informatics, College of Health Sciences, Saudi Electronic University, Jeddah 93499, Saudi Arabia; 3 Management Department, BINUS Online Learning, Bina Nusantara University, Jakarta 11480, Indonesia

We really appreciate the comments given by [1]. These comments are very valuable to us and will improve the value of our future research. It is necessary to know the background of our research in Saudi Arabia which has special characteristics related to the demographics in Saudi Arabia [2]. As many as 95% of Saudi Arabian citizens have internet access and more than 85% of citizens have an awareness of health applications in Saudi Arabia. Based on this data, Saudi Arabia has an above-average level of technology adoption compared to other countries.

A characteristic that cannot be ignored is the behavior of citizens in Saudi Arabia who are very submissive to the Kingdom of Saudi Arabia in many policies. A massive modernization is being carried out by the Kingdom of Saudi Arabia which makes Saudi Arabia an open country to the whole world. The COVID-19 Patient Tracing Policy and several other policies regulated by the Saudi Arabian government are well complied with by citizens [3]. In this study, we can see the high level of participation of respondents, particularly in answering and adopting several health applications made by the government [1].

We strongly agree that our research differs from [4] in terms of pandemic time and country context. In our opinion, this is not a barrier to using the Health Belief Model (HBM) in this study. The reason we use HBM compared to other models is because we think that HBM is the most appropriate model among others for measuring the adoption of health-related technologies. In our opinion, diffusion of innovation (DOI) lacks accuracy and specificity in health research. Why we did not include the dimensions of perceived susceptibility and severity is because in the preliminary study these two dimensions had low reliability and the respondents answered inconsistently and caused instrument bias. In the end, we decided not to use either dimension in the model. Our exclusion in terms of both perceived susceptibility and severity dimensions does not detract from the essence of this model. The focus of our research is on dimensions, so this exception will be a limitation of our research. In the field study that we found that it was not possible to include these two dimensions in the research model, so we did not force these two dimensions to be included in our research model.

This study was dominated by respondents aged 18–30 years, though the other age ranges had a small proportion, but we anticipated the accuracy of this study by using some preliminary questions such as “Are you accustomed to using health app on a smartphone?” We hope that this preliminary question can minimize misconceptions by old age respondents who do not have a close relationship to technology.

We really appreciate the comments provided by [1]. We hope that this research can be a reference and enrich knowledge, especially on the adoption of health technology for further research.

## Data Availability

Not applicable.
